# Targeting ryanodine receptors with allopurinol and xanthine derivatives for the treatment of cardiac and musculoskeletal weakness disorders

**DOI:** 10.1073/pnas.2422082122

**Published:** 2025-06-13

**Authors:** Marco C. Miotto, Estefania Luna-Figueroa, Carl Tchagou, Laith Bahlouli, Steven Reiken, Haikel Dridi, Yang Liu, Gunnar Weninger, Andrew R. Marks

**Affiliations:** ^a^Department of Physiology and Cellular Biophysics, Columbia University Vagelos College of Physicians and Surgeons, New York, NY 10032; ^b^Clyde and Helen Wu Center for Molecular Cardiology, Columbia University Medical Center, New York, NY 10032

**Keywords:** ryanodine receptor, calcium sensitizing, allopurinol, xanthine derivatives, cardiac and muscle weakness

## Abstract

Ryanodine receptors (RyRs) are intracellular Ca^2+^ channels essential for muscle contraction. Caffeine, a xanthine derivative, has been known for decades to increase muscle contraction and enhance activation of RyRs by increasing the sensitivity to Ca^2+^. We previously showed that xanthine, the only physiologically relevant xanthine derivative, also binds to and activates RyR2. Most xanthine derivatives and analogs are safe and widely prescribed, with the most popular being the xanthine oxidoreductase inhibitor allopurinol (~15M yearly prescriptions in USA). We propose that xanthine derivatives and analogs that enhance RyRs activity could be used for lead optimization and eventually for the treatment of the diseases that exhibit decreased muscle contraction and reduced RyRs activity, such as RyR1-related diseases, sarcopenia, and heart failure. Here, we show by cryo-EM that xanthine derivatives, analogs, and other related compounds bind to the xanthine/caffeine binding site and activate RyR1, and identify 4-oxopyrimidine as the minimal motif necessary for such interaction.

## Significance

Cardiac and skeletal muscle weakness are serious health problems resulting from hereditary or age-related diseases. We study the ryanodine receptors, proteins that play an essential role in cardiac and skeletal muscle contraction. Genetic variations in ryanodine receptors lead to debilitating and deadly diseases. Age-related changes in the expression or function of ryanodine receptors play a role in the development of heart failure and sarcopenia. Our aim is to develop drugs that bind to and improve the function of ryanodine receptors, resulting in improved cardiac and skeletal muscle contraction. Using cryo-EM, we determined the minimal chemical motif for compounds to bind to and activate ryanodine receptors, opening avenues to develop drugs for potential therapeutic use.

Ryanodine receptors (RyRs) are intracellular calcium channels involved in the excitation–contraction coupling in muscle cells and in calcium signaling in neurons and other cell types. RyR research has been mostly focused on genetic mutations or posttranslational modifications that render the channel leaky and lead to skeletal muscle (RyR1), cardiac (RyR2), or neurological (RyR1, RyR2, and RyR3) disorders (1, 2). Consequently, several RyRs inhibitors and stabilizers have been developed to prevent the pathological leak of sarcoplasmic reticulum’s calcium ions (Ca^2+^) (2, 3). However, there are no drugs for diseases involving downregulated RyRs (loss-of-function RyRs mutations or decreased expression/activity of RyRs). Downregulated RyRs have been found in diseases such as sarcopenia (aged-related muscle weakness with a prevalence >20% in older adults) (4–6), heart failure (aged-related cardiac weakness with a prevalence >5% in older adults) (7–11), intensive care unit (ICU) muscle weakness (prevalence of 33% of ICU patients) (12), myopathies (13), and rare hereditary conditions such as RyR1-related diseases (14) and Ca^2+^ release deficiency syndrome (15).

**Fig. 1.**
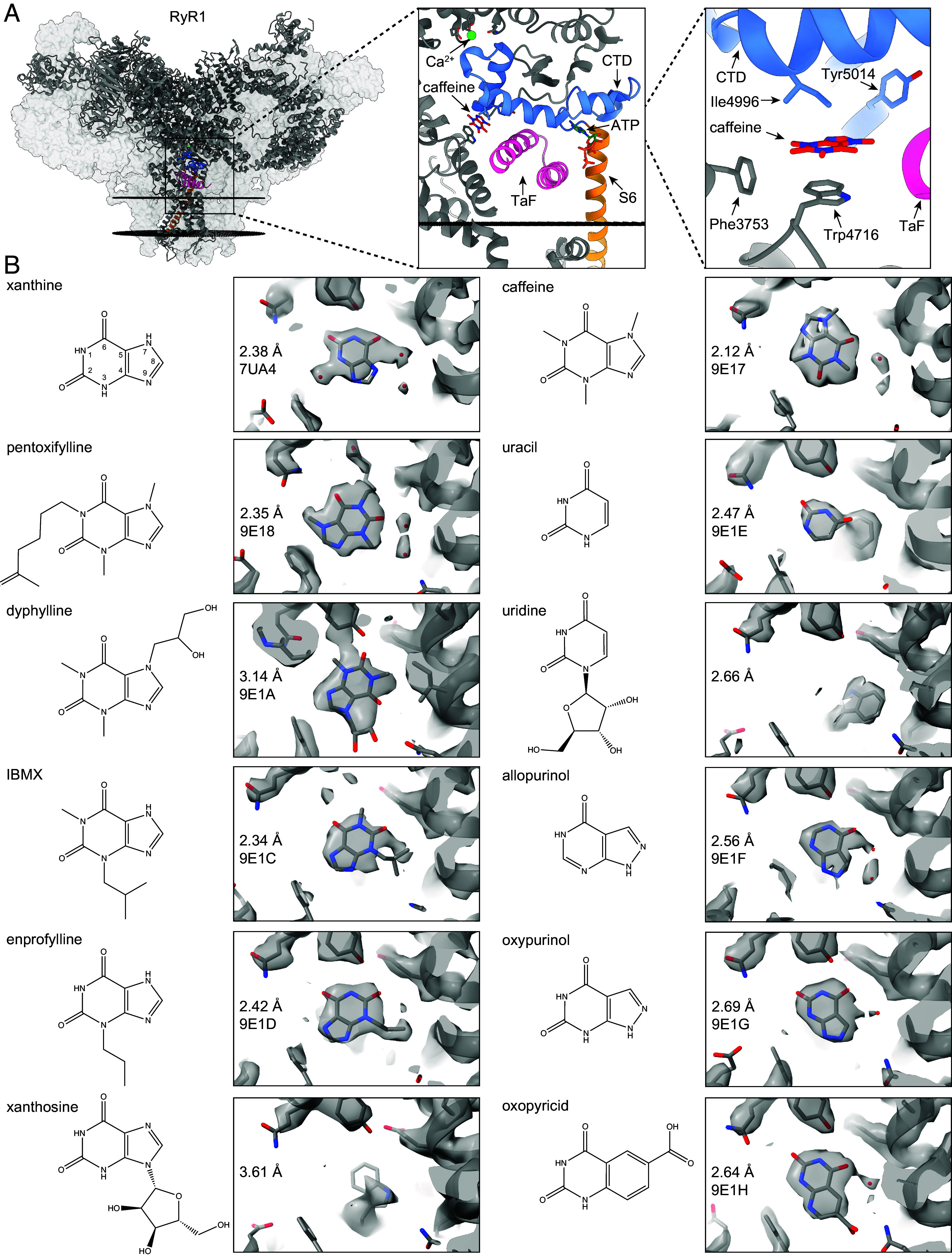
Cryo-EM of xanthine derivatives, analogs, and related compounds in the xanthine/caffeine binding site of RyRs. (*A*) Atomic model of RyR1 (PDB:9E17, gray), highlighting the location of Ca^2+^ (green), caffeine (red), ATP (dark green), the signal transducing domains CTD (blue) and TaF (pink), and the pore S6 helix (orange). To facilitate visualization, only the front protomer is shown, while the other three protomers are shown as transparent volumes. The sarcoplasmic reticulum membrane is shown as black discs. (*B*) For each compound, we show the chemical structure (*Left*) and the sharpen cryo-EM map (gray) overlapped with the respective atomic model (cartoon), local resolution achieved, and PDB identifier (*Right*). For clarity, the atom indexing is presented only for xanthine (inner numbers). All tested xanthine, allopurinol, and uracil derivatives bind to the xanthine/caffeine site except for xanthosine and uridine.

Caffeine, a xanthine derivative found in plants, has been used for decades as a pharmacologic research tool for activating RyRs (16), and its functional and binding properties have been extensively described (17–19). Caffeine interacts with RyR1–Trp4716 by pi–pi stacking in a hydrophobic pocket formed also by residues RyR1–Phe3753 and RyR1–Ile4996, as described by cryo-EM (20). This binding site is highly conserved among isoforms (RyR1, RyR2, and RyR3) and species, but its physiological role remains uncovered. Mechanistically, caffeine stabilizes the C-terminal domain (CTD) which is responsible for transducing the Ca^2+^ binding signal to the S6 pore helix, leading to gating (Fig. 1*A*). Since 1951, caffeine has been shown to enhance force and contractility of isolated skeletal muscles (21) and muscle cells in vitro (22). However, the millimolar concentrations needed to have a significant effect are toxic for human consumption, rendering caffeine of little therapeutic interest (23). Xanthine is a physiological metabolite of the purine catabolism, resulting from the activity of the enzyme xanthine oxidase which catalyzes the reaction: hypoxanthine → xanthine → uric acid. We showed that xanthine binds to the same conserved site in RyR2, interacting with residues RyR2–Trp4645 and RyR2–Ile4926 and potentially engaging RyR2–Tyr4944 (24). Xanthine activates RyR2 in the micromolar range, suggesting that it could be the endogenous ligand for this conserved binding site (24, 25). Unfortunately, the precise atomic description of the coordination mode of caffeine and xanthine remained unclear since the resolution achieved by cryo-EM was not sufficient to resolve the structure of these small and symmetric molecules. Other xanthine derivatives have been shown to activate RyRs in a similar way as caffeine, suggesting a permissive binding site (26). In particular, substituents in positions 1, 3, and 7 of the xanthine scaffold were shown to be more activating, while substituents in positions 8 and 9 were shown to be less activating compared to xanthine (26). However, the minimal motif for binding, necessary to design derivatives with high specificity and affinity for RyRs, has not been described.

Here, using cryo-EM, we explore multiple xanthine derivatives and related compounds and identify 4-oxopyrimidine/2-pyridone as the minimal motif necessary for binding to the xanthine/caffeine binding site in RyRs. While aromaticity is necessary for pi stacking with the Trp residue (RyR1–Trp4716 or RyR2–Trp4645), the N–H hydrogen is involved in hydrogen bonding with the Tyr residue (RyR1–Tyr5014 or RyR2–Tyr4944). Although the interaction with the Tyr residue is not necessary for the activation of RyRs, an additional hydrogen bond increases the binding strength allowing for concentrations of xanthine that are physiologically relevant to activate the channel. Allopurinol and oxypurinol, both of which contain this 4-oxopyrimidine motif, bind to the xanthine/caffeine site. Allopurinol/oxypurinol binding to RyRs could explain some of the observed beneficial muscular effects and be leveraged for the treatment of RyR-related diseases.

## Results

### Structural Binding Determinants of Xanthine Derivatives and Related Compounds.

**Fig. 2.**
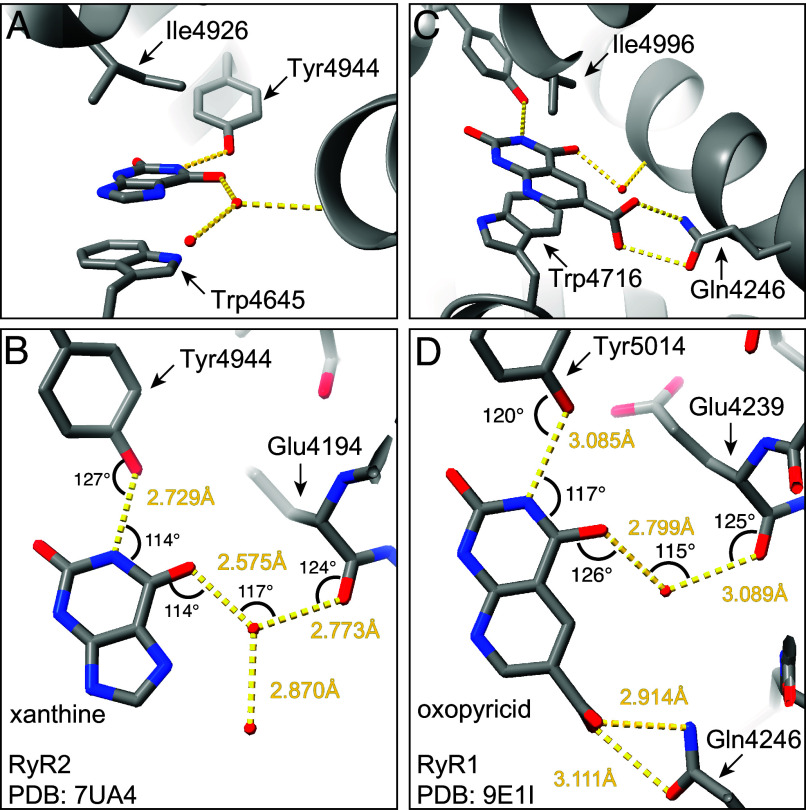
Coordination mode of xanthine and oxopyricid. (*A* and *B*) Coordination mode of xanthine in the xanthine/caffeine binding site of RyR2, showing pi stacking of xanthine with RyR2–Trp4645 (*A*), and hydrogen bonding with RyR2–Tyr4944 and RyR2–Glu4194, through a water bridge (*B*). Measured distances (yellow dashed lines) and angles (black curve lines) suggest a strong hydrogen bond network. (*C* and *D*) Coordination mode of oxopyricid in the xanthine/caffeine binding site of RyR1, showing pi stacking of oxopyricid with RyR1–Trp4716 (*C*), and hydrogen bonding with RyR1–Tyr5014, RyR1–Gln4246, and RyR1–Glu4239, through a water bridge (*D*). Measured distances (yellow dashed lines) and angles (black curve lines) suggest a strong hydrogen bond network.

In order to determine the coordination mode of xanthine and related compounds, we first reprocessed the best available RyR2 dataset in the presence of xanthine (EMPIAR-11403, PDB 7UA4). Using an improved local refinement strategy focused on the xanthine/caffeine binding site, we reached a local resolution of 2.38 Å (Fig. 1*B* and *SI Appendix*, Fig. S1). This resolution level allowed us to confidently place the xanthine molecule and three water molecules of the hydration shell (Fig. 2 *A* and *B*). We observed that xanthine sits between aromatic RyR2–Trp4645 (pi stacking) and hydrophobic RyR2–Ile4926, forming a hydrogen bond with RyR2–Tyr4944 (through imide N1-H), and interacting with the backbone carbonyl of RyR2–Glu4194 through a bridge water molecule (through carbonyl C6=O, Fig. 2*B*). We also reprocessed the best available RyR1 dataset in the presence of caffeine (EMPIAR-10997, PDB 9E17) using our focused strategy, which led to a local resolution of 2.12 Å (Fig. 1 and *SI Appendix*, Fig. S1). This resolution allowed us to confidently model the orientation and position of the caffeine molecule in the binding site, which was unclear in the previous model. We observed that caffeine is as expected pi stacked between RyR1–Trp4716 and RyR1–Ile4996 and interacts with the backbone carbonyl of RyR1–Glu4239 through a bridge water molecule (through carbonyl C_6_=O) but does not engage RyR1–Tyr5014 (the N1 is methylated). The small size of xanthine and caffeine molecules in addition to the hydration shell is probably what in the past had hindered the determination of the coordination of caffeine, and now with better processing power and resolution, we can be certain of the binding mode of both xanthine and caffeine. Moreover, engagement of RyR2–Glu4194 and RyR1–Glu4239 suggests that stabilization of the thumb and forefinger domain (TaF) adds another layer of signal transduction (Fig. 1*A*). Interestingly, the position of caffeine is flipped compared to that of xanthine, suggesting a flexible binding site adaptable to chemical substitutions.

To determine the minimal motif necessary for binding to the xanthine/caffeine binding site and how different substitutions affect the coordination mode, we resolved the cryo-EM structure of RyR1 in the presence of different xanthine derivates. Cryo-EM data collection and processing statistics are summarized in *SI Appendix*, Table S1. A representative micrograph, 2D class averages, and 3D reconstruction, and data processing workflows are described in Methods and shown in *SI Appendix*, Figs. S1 and S2. We analyzed the following xanthine derivatives with substituents in different positions: the commercially available adenosine receptor antagonists and phosphodiesterase inhibitors pentoxifylline (PDB 9E18, 9E19), dyphylline (PDB 9E1A, 9E1B), IBMX (PDB 9E1C), and enprofylline (PDB 9E1D) and the naturally occurring nucleoside xanthosine (Fig. 1*B* and *SI Appendix*, Fig. S1). Using our focused cryo-EM processing, we achieved local resolutions in the 2.34 to 3.14 Å range. We found a clear cryo-EM density in the xanthine/caffeine site for pentoxifylline, dyphylline, IBMX, and enprofylline, while we found no density for xanthosine (Fig. 1*B*). We were able to model the most likely position of the xanthine derivates thanks to the clear additional densities from the substituent groups and found the xanthine scaffold in a similar orientation in all four samples, stacked on RyR1–Trp4716. In the case of xanthosine, the large off-plane sugar substituent at position N9 would crash with residue RyR1–Phe3753 preventing binding, in agreement with substitutions at position 9 being less activating (26). Second, we found that RyR1–Tyr5014 was not engaged when N1 was methylated or substituted (IBMX and pentoxifylline) but it was engaged when N1 was unmodified or protonated (enprofylline). In the case of dyphylline, RyR1–Tyr5014 would seem to be engaged interacting with carbonyl C2=O, but the lower resolution of this dataset makes it unclear. Since we have found RyR1 particles in the open state with RyR1–Tyr5014 in the unengaged conformation (pentoxifylline), we concluded that this conformational flexibility of RyR1–Tyr5014 is not necessary for gating, but it might contribute to the strength of the interaction with xanthines by involvement of an additional hydrogen bond. Additionally, for pentoxifylline, we observed that the carbonyl C2=O interacts with the backbone carbonyl of residue RyR1–Glu4239 through a water molecule as seen previously for xanthine and caffeine. From this analysis, we concluded that aromaticity is necessary for pi stacking with RyR1–Trp4716, the protonated N1 (unmodified) is necessary to engage RyR1–Tyr5014, and the carbonyl C2=O or C6=O is necessary for interacting with the backbone carbonyl of RyR1–Glu4239 through a water molecule. The imidazole 5-member ring does not play an important role besides providing more surface for pi stacking with RyR1–Trp4716, suggesting the unmodified six-member ring of xanthine (2,4-dioxopyrimidine) could be the minimal motif for binding.

To support the hypothesis that 2,4-dioxopyrimidine is the minimal binding motif, we resolved the structure of RyR1 in the presence of uracil (PDB 9E1E) and its sugar-substituted form uridine, the xanthine oxidase inhibitors allopurinol (hypoxanthine analog, PDB 9E1E) and oxypurinol (xanthine analog, PDB 9E1G), and oxopyricid (an unrelated 2,4-dioxopyrimidine diaromatic derivative, PDB 9E1H, 9E1I) by cryo-EM (Fig. 1*B* and *SI Appendix*, Fig. S1). We found that uracil, but not uridine, binds to the xanthine/caffeine site, confirming that the six-membered ring of xanthine is sufficient for the interaction and that the large ribose substituent would clash with RyR1–Phe3753. Allopurinol and oxypurinol, two clinically relevant xanthine analogs, also bind in a mode almost identical to xanthine. Interestingly, allopurinol, which contains only the C6=O carbonyl, is able to interact with the backbone carbonyl of RyR1–Glu4239 through a shell water molecule, suggesting the minimum motif for binding could be 4-oxopyrimidine, which contains only the C6=O carbonyl. Since the pyrimidine N3 has no important role in the interaction, we also propose that 2-pyridone (N3 is replaced by C3) could be a minimal motif for binding. Finally, our rational discovery of compounds led to oxopyricid which also binds to the xanthine/caffeine site. Surprisingly, oxopyricid led to more RyR1 particles in the open state (70% particles in the open state) than the other water-soluble compounds (5% for pentoxifylline, 15% for dyphylline, and 20 to 30% for caffeine). The remaining compounds required 2% DMSO for solubility purposes and all particles were found in the primed state. This suggests that DMSO prevents transition to the open state. Additionally, oxopyricid contains a carboxylic motif at C8 that not only makes it highly water soluble but also able to interact with RyR1–Gln4246 through one or two hydrogen bonds, further engaging the TaF domain (Fig. 2 *C* and *D*). The additional hydrogen bond(s) strengthen the interaction, probably increasing the residence time (1/k_off_) of oxopyricid, compared to the other xanthine derivatives. Because the conformation of the xanthine/caffeine binding site remains unchanged independently of ligand bound and state (*SI Appendix*, Fig. S3), the residence time of the ligands would be the main factor that facilitates the slow transition from the primed to the open state.

### Xanthine Derivatives and Related Compounds Increase Ryanodine Binding and Muscle force.

**Fig. 3.**
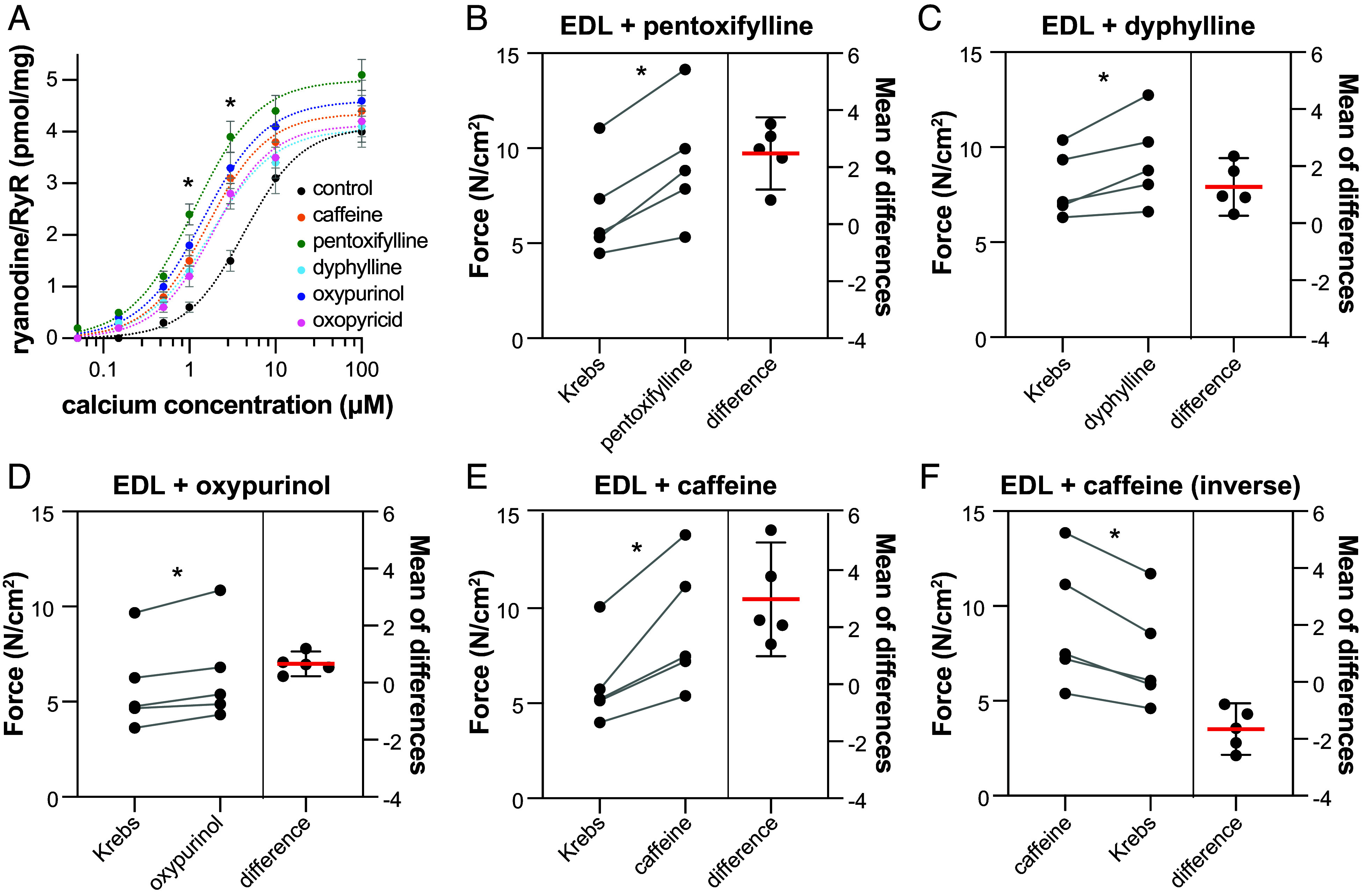
Xanthine derivatives and related compounds increase ryanodine binding and muscle twitch force. (*A*) Ryanodine binding assay of rabbit RyR1 microsomes in the presence of 1 mM of caffeine, pentoxifylline, dyphylline, oxypurinol, or oxopyricid at increasing Ca^2+^ free concentrations (50 nM, 150 nM, 500 nM, 1 µM, 3 µM, 10 µM, and 100 µM). For 1 µM and 3 µM Ca^2+^ free concentrations, all compounds showed significant difference with the control (*t* test, *P < 0.05, n = 2). For visualization purposes, a sigmoidal curve was fitted for each compound dataset (dotted line). (*B*–*E*) EDL twitch force measurement before and after a 5-min incubation with Krebs buffer in the presence of 5 mM of pentoxifylline (*B*), dyphylline (*C*), oxypurinol (*D*), or caffeine (*E*). Using a paired *t* test, EDL muscles showed significant increase in twitch force in the presence of pentoxifylline (*P* = 0.006, mean difference=2.48, n = 5), dyphylline (*P* = 0.025, mean difference = 1.27, n = 5), oxypurinol (*P* = 0.013, mean difference = 0.66, n = 5), or caffeine (*P* = 0.014, mean difference = 2.98, n = 5). (*F*) EDL twitch force measurement before and after a 5-min incubation with Krebs buffer (wash) of EDL preincubated with caffeine. Using a paired *t* test, EDL muscles showed significant decrease in twitch force after the wash (*P* = 0.007, mean difference = −1.65, n = 5).

We wondered whether the compounds binding to the xanthine/caffeine binding site had similar functional effects compared to caffeine. First, we measured the Ca^2+^-dependent ryanodine binding using a [^3^H] ryanodine binding assay, which estimates the percentage of channels that shift to the open state. We observed that 1 mM caffeine, pentoxifylline, dyphylline, oxypurinol, and oxopyricid all had similar effects on the Ca^2+^-dependent ryanodine binding of RyR1 (Fig. 3*A*). We observed a significant increase of activation of RyR1 at 1 µM and 3 µM free Ca^2+^ for all compounds compared to controls (*P* < 0.05). This suggests that these compounds are RyR1 activity enhancers by increasing the sensitivity to Ca^2+^, as described for caffeine.

Caffeine has been reported to increase twitch force of skeletal muscle by enhancing RyR1 activity (21). We performed ex vivo twitch force experiments on extensor digitorum longus (EDL) muscles from mice. We measured the twitch force before and after a 5-min incubation with 5 mM caffeine, pentoxifylline, dyphylline, and oxypurinol (Fig. 3 *B*–*E*). We found that incubation with these compounds significantly increased the twitch force of EDL muscles after 5 min of incubation (*P* < 0.05). This short incubation aimed to minimize off-target activity that could confound our results (pentoxifylline and dyphylline are phosphodiesterase inhibitors and oxypurinol is a xanthine oxidase inhibitor). Moreover, the inverse experiment, a 5-min wash with Krebs buffer of the EDL muscles preincubated with caffeine, led to a significant decrease of twitch force, suggesting reversibility (Fig. 3*F*). This agrees with these compounds binding and enhancing RyR1 activity in a direct and reversible manner, reassuring that the compounds alter muscle function as hypothesized.

## Discussion

**Fig. 4.**
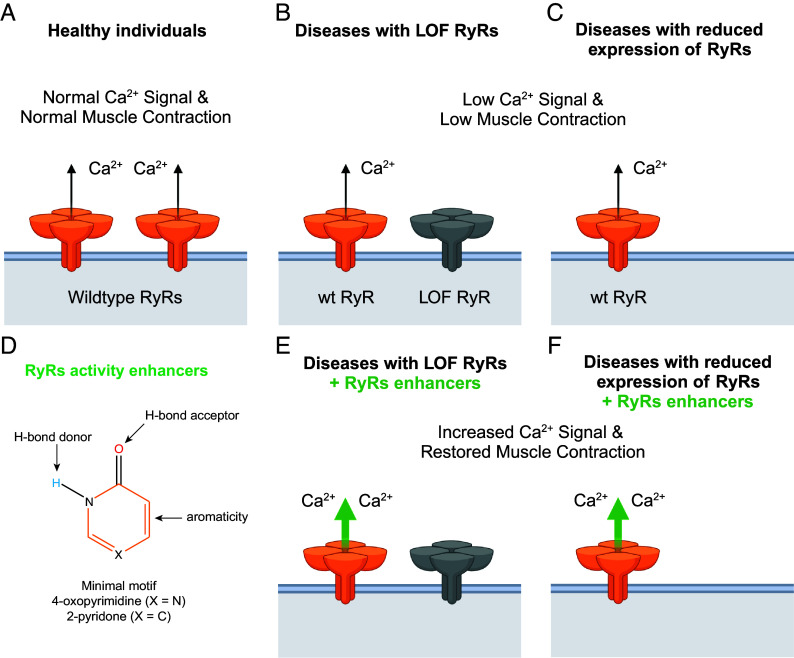
Ryanodine receptors activity enhancers as potential therapeutics. (*A*–*C*) Schematic representation of RyRs and muscle contraction in healthy conditions (*A*), in diseases involving loss-of-function RyRs mutations (*B*), and in diseases involving reduced expression of RyRs (*C*). (*D*) Minimal motif necessary for compounds to bind to the xanthine/caffeine site of RyRs and to be used as a scaffold to develop specific RyRs activity enhancers. (*E* and *F*) Schematic representation of RyRs and muscle contraction in the presence of RyRs activity enhancers in diseases involving loss-of-function RyRs mutations (*E*) and in diseases involving reduced expression of RyRs (*F*).

In healthy individuals, the activity of RyRs is finely regulated, controlled both by posttranslational modifications and expression levels of RyRs (Fig. 4*A*). Dysregulation of RyRs activity has been shown to be detrimental for the normal function of muscle cells and health of individuals. Destabilization of the RyR closed state, either due to stress-induced posttranslational modifications or gain-of-function mutations, leads to leaky RyRs and results in disease states that can be lethal (2). Downregulation of RyRs activity leads to calcium deficiency and muscle weakness disorders (Fig. 4 *B* and *C*) (13–15). Downregulation of RyRs activity has been found in diseases involving loss-of-function mutations (RyR1-related diseases and RyR2 calcium release deficiency syndrome) and reduced expression of RyRs (myopathies, sarcopenia, heart failure, cardiomyopathies, ICU muscle weakness, and others) (13–15). Pharmacologically enhancing RyRs activity is a potential therapeutic approach to treat these disorders. Caffeine has been known for decades to enhance the activity of RyRs (16, 17, 21), but the mode of binding has been elusive. Recent advances in cryo-EM hardware and software allowed us now to routinely achieve 2 to 3 Å resolution and to confidently determine the mode of binding of caffeine to RyR1, xanthine to RyR2, and other related compounds to RyR1. Interestingly, the conformation of the xanthine/caffeine site remained unaltered independently of ligand binding, RyR isoform (RyR1 vs. RyR2 vs. RyR3), and state (primed vs. open) further confirming the high conservation of this site (*SI Appendix*, Fig. S3). By analyzing the modes of binding of xanthine derivatives and analogs, we postulate that the 4-oxopyrimidine/2-pyridone motif is sufficient and necessary for binding to the xanthine/caffeine site in RyRs (Fig. 4*D*). The 4-oxopyrimidine/2-pyridone motif engages Trp4716, Tyr5014, and Glu4239 in RyR1 and Trp4645, Tyr4944, and Glu4194 in RyR2. The interaction with these residues would stabilize the CTD and TaF domains, allowing for better signal transduction from Ca^2+^ binding to pore gating. The proposed motif agrees with the inability of adenine and its derivatives to bind to this site, since adenine N1 is not protonated being unable to form a hydrogen bond with RyR1–Tyr5014 and there is no carbonyl that could act as a strong hydrogen bond acceptor to interact with RyR1–Glu4239 through a water bridge (27). Moreover, the proposed 4-oxopyrimidine motif allowed us to rationally find the nonpurine compound oxopyricid that binds to the xanthine/caffeine binding site and additionally engages RyR1–Gln4246 leading to a more stable complex and more open particles.

Development of 4-oxopyrimidine/2-pyridone derivatives that specifically bind to the xanthine/caffeine site could be exploited to enhance the activity of RyRs in diseases related to loss-of-function RyRs mutations (Fig. 4*E*) or reduced expression of RyRs (Fig. 4*F*). RyRs are also expressed in neurons and lymphocytes, suggesting that modulation of RyRs could potentially help in the treatment of neurological and immunological disorders. Unlike xanthine derivatives and analogs, development of 4-oxopyrimidine/2-pyridone derivatives would retain the RyRs activating properties while decreasing binding to other targets such as xanthine oxidoreductase, phosphodiesterase, and adenosine receptors. The advantage of 4-oxopyrimidine/2-pyridone derivatives is that, by binding to the xanthine/caffeine site, they increase Ca^2+^ sensitivity of RyRs retaining the functional and temporal activity of normal RyRs. Other activators and agonists independent of Ca^2+^ could chronically activate RyRs thereby enhancing pathologic Ca^2+^ leak, which is associated with gain-of-function RyR diseases such as Malignant Hyperthermia and Catecholaminergic Polymorphic Ventricular Tachycardia.

Finally, several clinical trials have been carried out to assess the potential of allopurinol/oxypurinol to treat cardiac diseases. While a meta-analysis showed significant cardioprotective effects of allopurinol (28), other studies showed no improvement (29) or improvement only at higher doses of allopurinol (30–32). Another meta-analysis comparing purine xanthine oxidase inhibitors (allopurinol/oxypurinol) to nonpurine xanthine oxidase inhibitors (febuxostat/topiroxostat) showed that only the former had cardioprotective effects (33), suggesting that inhibiting xanthine oxidase was not sufficient and a secondary mechanism was probably present. The main weakness of these studies is the established paradigm, which is that inhibition of xanthine oxidase, lowering uric acid, and preventing oxidative damage are responsible for all the cardioprotective effects. The present study shows that allopurinol/oxypurinol binds to the xanthine/caffeine site of RyRs, suggesting that allopurinol/oxypurinol could enhance RyRs activity and be beneficial in disorders with downregulated RyRs. The paradigm that the cardioprotective effect of allopurinol/oxypurinol arises only from xanthine oxidase inhibition might need to be revisited to include RyRs as targets of allopurinol/oxypurinol. Similarly, it becomes important to consider RyRs as potential off-targets of xanthine derivatives (pentoxifylline, dyphylline, IBMX, and enprofylline).

## Materials and Methods

### Chemicals and Reagents.

Pentoxifylline (3,7-dimethyl-1-(5-oxohexyl)xanthine), dyphylline (1,3-dimethyl-7-(2,3-dihydroxypropyl)xanthine), IBMX (3-iso-1-methylxanthine), enprofylline (3-propylxanthine), xanthosine, uracil, uridine, oxypurinol, and allopurinol were purchased from Sigma-Aldrich. Oxopyricid, which we coined as the name for 2,4-dioxo-1H,2H,3H,4H-pyrido[2,3-d]pyrimidine-6-carboxylic acid, was purchased from ChemCruz Biochemicals (sc-343457), a division of Santa Cruz Biotechnology. All reagents were purchased from Sigma-Aldrich unless stated otherwise.

### Purification of Endogenous RyR1 from Rabbit Skeletal Muscle.

RyR1 was purified from rabbit skeletal muscle using a protocol previously reported by our lab (19, 34). All purification steps were performed on ice unless otherwise stated. Rabbit back and thigh muscle tissue was harvested and snap frozen in liquid nitrogen immediately after rabbit was killed prior to shipping on dry ice and storage at −80 °C (BioIVT). Then, 20 g of skeletal muscle tissue was resuspended in 150 mL buffer A (10 mM tris maleate pH 6.8, 1 mM EGTA, 1 mM benzamidine hydrochloride, 0.5 mM AEBSF) and homogenized in a Waring blender for 3 min. The suspension was centrifugated for 10 min at 11,000×*g* and the supernatant was filtered through cheesecloth to remove debris. After centrifugation for 30 min at 36,000×*g*, the pelleted membrane fraction was solubilized in 8 mL of buffer B [10 mM HEPES pH 7.4, 0.8 M NaCl, 1% CHAPS, 0.1% phosphatidylcholine, 1 mM EGTA, 2 mM DTT, 0.5 mM AEBSF, 1 mM benzamidine hydrochloride, one protease inhibitor tablet (Pierce)] followed by homogenization using a glass tissue grinder (Kontes). The homogenate was diluted with buffer C (buffer B without NaCl) at a 1:1 ratio following 10 more strokes of homogenization. After centrifugation for 30 min at 100,000×*g* to remove CHAPS-insoluble material, the supernatant was filtered (0.22 µm) and then loaded onto a HiTrap Q HP column (1 mL, GE Healthcare) previously equilibrated with buffer D [10 mM HEPES pH 7.4, 400 mM NaCl, 0.2% CHAPS, 1 mM EGTA, 0.5 mM TCEP, 0.01% 1,2-dioleoyl-sn-glycero-3-phosphocholine (DOPC, Avanti), 0.5 mM AEBSF, and 1 mM benzamidine hydrochloride]. After washing with six column volumes of buffer D, the immobilized protein on the HiTrap Q HP column was eluted with a linear gradient from 480 to 520 mM NaCl using a linear mix of buffer D and buffer E (buffer D with 600 mM NaCl). RyR1-containing fractions were pooled and concentrated to ~10 g/ L (20 to 40 µL) using 100,000 kDa cut-off centrifugation filters (MilliporeSigma).

### Cryo-EM Sample Preparation and Data Collection.

Cryo-EM sample preparation and data collection were performed according to our lab protocols (34, 35). Cryo-EM samples were prepared by adding 10 mM NaATP, 30 μM free Ca^2+^ (1.6 mM total Ca^2+^), and 2 mM of compounds to 10 g/L RyR1 aliquots. Compound stock solutions were prepared at 20 mM in buffer D for pentoxifylline, dyphylline, and oxopyricid (2,4-dioxo-1H,2H,3H,4H-pyrido[2,3-d]pyrimidine-6-carboxylic acid) and 100 mM in 100% DMSO for IBMX, enprofylline, xanthosine, allopurinol, oxypurinol, uracil, and uridine. Therefore, cryo-EM samples with IBMX, enprofylline, xanthosine, allopurinol, oxypurinol, uracil, uridine contain 2% DMSO. Free Ca^2+^ concentrations were calculated using MaxChelator. The final sample (3 μL) was applied to UltrAuFoil holey gold grids (Quantifoil R 0.6/1.0, Au 300) previously cleaned with easiGlow (PELCO). Grids were blotted with ashless filter paper (Whatman) using blot force 10 and blot time 8 s before plunge-freezing into liquid ethane chilled with liquid nitrogen using Vitrobot Mark IV (Thermo Fisher Scientific) operated at 4 °C with 100% relative humidity. High-resolution data collection was performed at Columbia University on a Titan Krios 300-kV (Thermo Fisher Scientific) microscope equipped with an energy filter (slit width 20 eV) and a K3 direct electron detector (Gatan). Data were collected using Leginon (36) and at a nominal magnification of ×105,000 in electron counting mode, corresponding to a pixel size of 0.83 Å. The electron dose rate was set to 16 e^−^/pixel per second with 2.5 s exposures for a total dose of 58 e/Å^2^.

### Cryo-EM Data Processing and Model Building.

Cryo-EM data processing and model building were adapted from our lab protocols (34, 35). Cryo-EM data processing was performed using cryoSPARC (37) with image stacks aligned using Patch motion and defocus value estimation by Patch CTF estimation (*SI Appendix*, Fig. S2). Particle picking was performed using Topaz trained with preexisting cryo-EM picked particles. Particles were subjected to a series of 2D classification, ab initio 3D reconstruction, homogeneous refinement, and heterogeneous refinement to separate good particles from bad particles and contaminants. Using a “pore” mask comprising the CSol + TaF + TM + CTD domains (residues 3660 to 5037), a local refinement followed by a local 3D classification was performed to further separate particles in the closed/primed state from those in the open state (*SI Appendix*, Fig. S2). Nonuniform refinement was performed to estimate global resolution of clustered classes (primed or open states). C4 symmetry expansion was performed before a second round of local refinement. The final local refinement was performed using a “small” mask comprising the xanthine/caffeine binding site. Local refinements used a “dynamic” mask parameter with a “mask far” value of 10 and 50 Å for the pore mask and the small mask, respectively. The calibration of the voxel size was performed using correlation coefficients with a map generated from the crystal structure of the NTD of RyR1 (2XOA) (38). The voxel size was altered by 0.005 Å per step, up to 20 steps in each direction. The initial model was RyR1 (PDB:7TZC). Model fittings and model building were performed in Coot (39), and final models were refined with Phenix tool RealSpaceRefine (40). Cryo-EM statistics are summarized in *SI Appendix*, Table S1. Figures of the structural analyses were created using ChimeraX (41) and Adobe Illustrator 2024.

### Ryanodine Binding Assay.

Skeletal muscle microsomes were prepared by homogenizing rabbit muscle using a blender with 200 mL of lysis buffer (20 mM Tris-maleate pH 7.4, 1 mM EDTA, 1 mM DTT, and Complete protease inhibitors). Homogenate was then centrifuged at 4,000×*g* for 15 min at 4 °C and the following supernatant was centrifuged at 50,000×*g* for 45 min at 4 °C. Pellets were resuspended in resuspension buffer (20 mM Tris-maleate, 300 mM sucrose, pH 7.4). Then, 50 mg of microsomes were diluted in 0.1 mL of ryanodine binding buffer (10 mM Tris‐HCl, pH 6.8, 1 M NaCl, and Complete protease inhibitors). Next, 0.1 mL of binding buffer was added so that the final reactions contained 20 nM [^3^H] ryanodine and calcium concentrations ranging from 50 to 100,000 nM. Reactions were done in duplicate in the absence (control) or presence of 1 mM caffeine, 1 mM oxypurinol, 1 mM oxopyricid, 1 mM pentoxifylline, or 1 mM dyphylline. Samples were incubated for 2 h at 37 °C and filtered through Whatman GF/B membrane filters presoaked with 1% polyethyleneimine in washing buffer. Filters were washed three times with 5 mL of washing buffer. The radioactivity remaining on the filters is determined by liquid scintillation counting to obtain bound [^3^H] ryanodine. Nonspecific binding was determined in the presence of 100‐fold excess of non‐labeled ryanodine. To plot the data and assess statistical significance, multiple two-tailed unpaired *t* tests were performed using GraphPad Prism 10.

### Twitch force Measurement.

Mice were euthanized using CO_2_ overdose followed by cervical dislocation, in accordance to the protocol #AC-AABP1551 approved by Columbia University’s Institutional Animal Care and Use Committee (IACUC), consistent with the AVMA Guidelines on Euthanasia (2020 edition). The extensor digitorum longus (EDL) muscles were dissected according to our protocols and isometric contractile properties characteristics were determined as described previously (42). The dissected EDL muscles were mounted to a force transducer and immersed in a tissue bath chamber filled with Krebs-Ringer buffer at 28 °C and oxygenated with a 95/5% mixture of O_2_/CO_2_. Each muscle was incubated in the solution for 10 min to allow the muscle to stabilize under the solution and adjusted to its optimal length to achieve the maximal twitch force. The EDL muscles were stimulated using square wave pulses for 600 ms at 1 Hz, using a Grass S48 stimulator from Grass Instruments, Aurora Scientific Inc, Aurora, ON, Canada. After the initial stimulation, a second stimulation was applied to the muscle after a 1-min recovery period. The average of the two measurements was plotted and used for statistical comparisons. The Krebs-Ringer buffer was removed and replaced with a Krebs-Ringer buffer containing 5 mM of pentoxifylline, dyphylline, oxypurinol, or caffeine. The muscles were then incubated for 5 min and stimulated as described before, followed by a second stimulation after 1 min. To prove reversibility, the Krebs-Ringer buffer containing 5 mM of caffeine was further removed and replaced with a Krebs-Ringer buffer (wash). As before, the EDL muscles were stimulated after 5 and 5 + 1 min. After measuring the contractile force, the length of the muscle was measured, and then it was dried and weighed. The contractile force production was normalized to the total cross-sectional area of the muscle strip and expressed in N/cm^2^. The cross-sectional area was calculated by dividing the muscle weight by its length and tissue density (1.056 g/cm^3^). Each condition was repeated with 5 EDL muscles. To plot the data and assess statistical significance, a two-tailed paired *t* test was performed using GraphPad Prism 10.

## Supplementary Material

Appendix 01 (PDF)

## Data Availability

Atomic models and cryo-EM maps are publicly available at wwPDB and EMDB under the following accession codes, respectively: 7UA4 (43) and EMD-47336 (44) (RyR2 in the open state + xanthine); 9E17 (45) and EMD-47357 (46) (RyR1 in the primed state + caffeine); 9E18 (47), EMD-47385 (48), and EMD-47396 (49) (RyR1 in the primed state + pentoxifylline); 9E19 (50), EMD-47386 (51), and EMD-47397 (52) (RyR1 in the open state + pentoxifylline); 9E1A (53), EMD-47387 (54), and EMD-47398 (55) (RyR1 in the primed state + dyphylline); 9E1B (56), EMD-47388 (57), and EMD-47399 (58) (RyR1 in the open state + dyphylline); 9E1C (59), EMD-47389 (60), and EMD-47400 (61) (RyR1 in the primed state + IBMX); 9E1D (62), EMD-47390 (63), and EMD-47401 (64) (RyR1 in the primed state + enprofylline); 9E1E (65), EMD-47391 (66), and EMD-47402 (67) (RyR1 in the primed state + uracil); 9E1F (68), EMD-47392 (69), and EMD-47403 (70) (RyR1 in the primed state + allopurinol); 9E1G (71), EMD-47393 (72), and EMD-47404 (73) (RyR1 in the primed state + oxypurinol); 9E1H (74), EMD-47394 (75), and EMD-47405 76) (RyR1 in the primed state + oxopyricid); and 9E1I (77), EMD-47395 (78), and EMD-47406 (79) (RyR1 in the open state + oxopyricid).
