# Supplementary material for: Targeting ryanodine receptors with allopurinol and xanthine derivatives for the treatment of cardiac and musculoskeletal weakness disorders
Source: Proc Natl Acad Sci U S A. 2025 Jun 13;122(24):e2422082122. doi: 10.1073/pnas.2422082122 (PMC12184490; doi:10.1073/pnas.2422082122)
Supplement: Supplementary file 1 — Appendix 01 (PDF) [file pnas.2422082122.sapp.pdf]

Supporting information

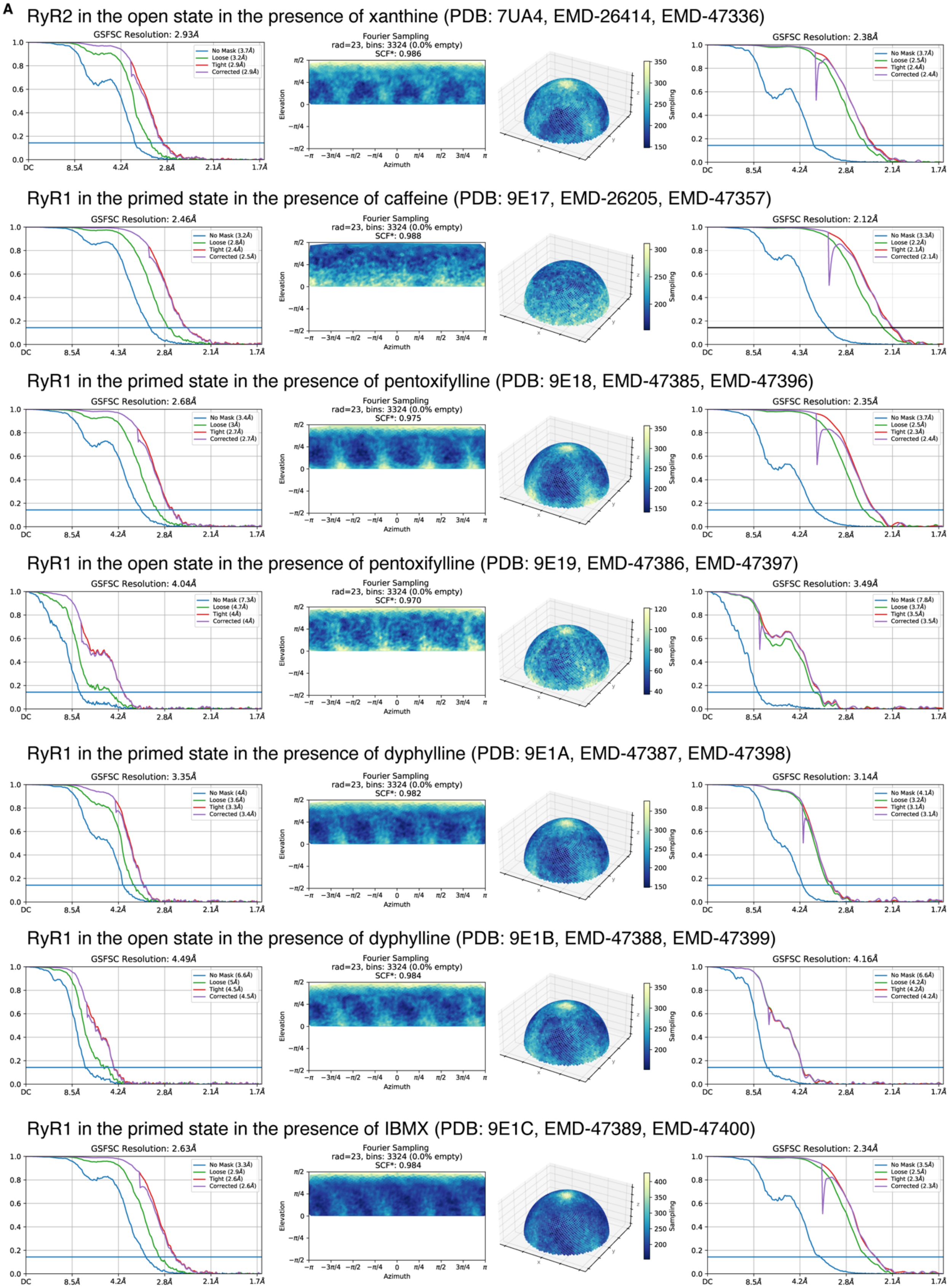

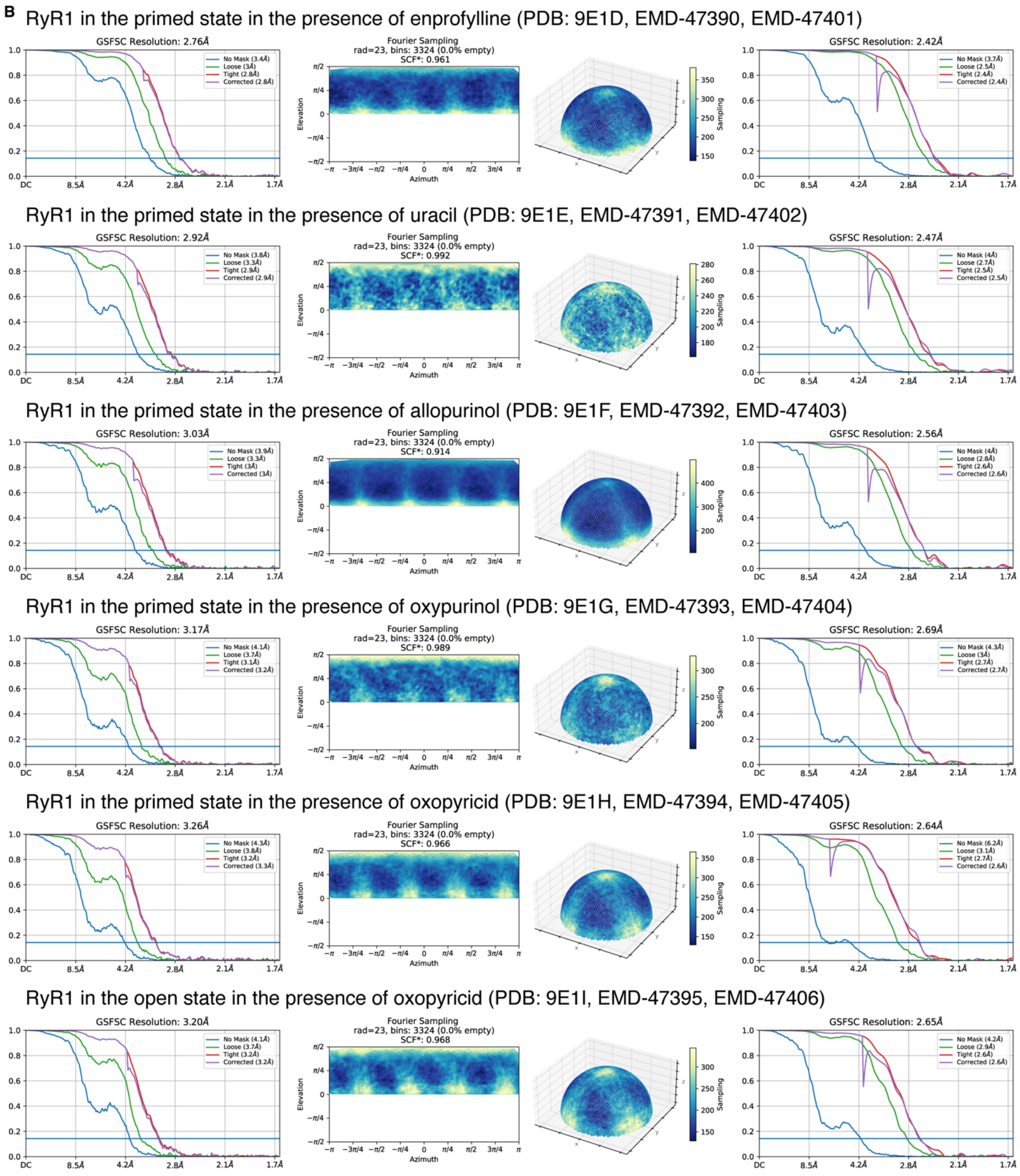

Figure S1. FSC curves and orientation diagnosis. A-B. The GSFSCs (left) and the Fourier sampling (middle) of the non-uniform refinement performed in cryoSPARC and the GSFSCs of the local refinement (right) for each structure are shown. SCF Values above 0.81 generally indicate adequate sampling.

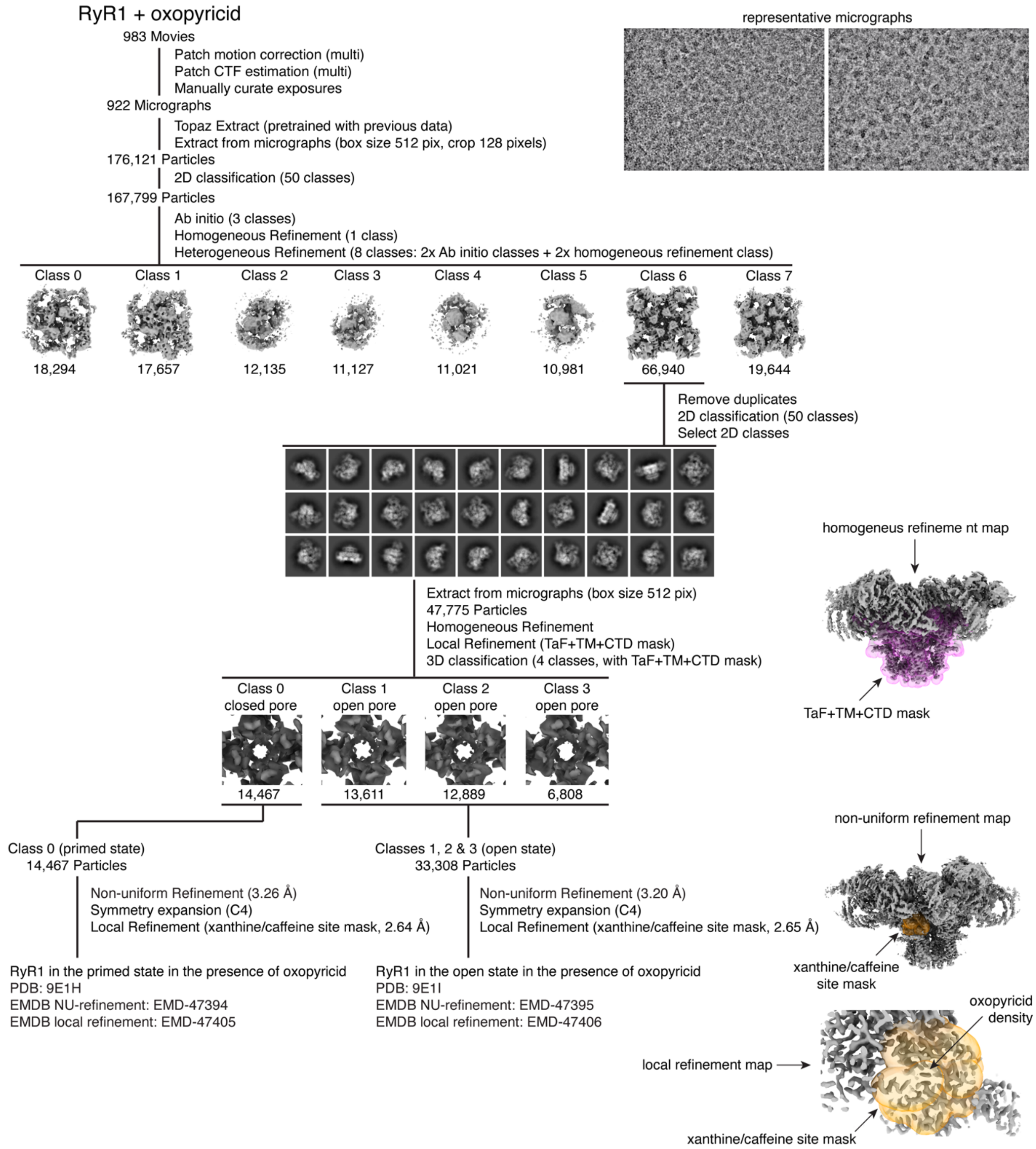

Figure S2. Cryo-EM data processing workflow. Entire cryoSPARC processing for the RyR1 + oxopyricid dataset to obtain the global and local refinement maps in the primed and open states. The number of particles and the resolution achieved for each refinement are shown for each step. Representative micrographs show good coverage with particles (top right). The global refinement map with the TaF+TM+CTD mask (right) and the local refinement map with the xanthine/caffeine site mask (bottom right) depict processing strategy. The same processing workflow was utilized for all datasets.

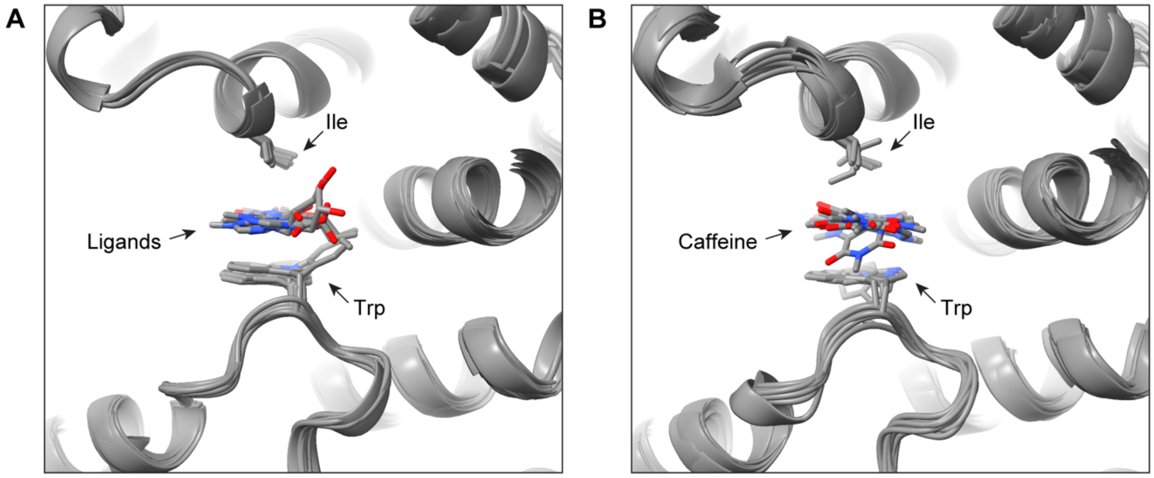

Figure S3. Conserved conformation of the xanthine/caffeine binding site. A. Overlapped atomic models of all structures presented in this paper (PDB 7UA4, 9E17, 9E18, 9E19, 9E1A, 9E1B, 9E1C, 9E1D, 9E1E, 9E1F, 9E1G, 9E1H, 9E1I) showing that the conformation of the xanthine/caffeine binding site, especially the backbone, remains mostly unchanged independently of ligand bound, RyR isoform (RyR1 vs RyR2), and state (primed vs open). **B.** Overlapped atomic models of structures in the presence of caffeine of pig primed RyR2 (PDB 6JHN), pig open RyR2 with CaM (PDB 6JRR), rabbit primed RyR1 in lipidic membranes (PDB 7M6A), rabbit open RyR1 with IpCa (PDB 8DVE), rabbit primed RyR1 in nanodiscs (PDB 8RRX), mouse primed RyR1 (PDB 8VJK), chimeric rabbit-insect open RyR1 (PDB 8XLF), mink open RyR3 (PDB 9C1F), and rabbit primed RyR1 (PDB 9E17). This shows that the conformation of the xanthine/caffeine binding site, especially the backbone, remains mostly unchanged independently of species (pig, mouse, rabbit, mink), RyR isoform (RyR1 vs RyR2 vs RyR3) and state (primed vs open).

Table S1. Cryo-EM Statistics.

**Sample**	**RyR1 + pentoxifylline**		**RyR1 + dyphylline**		**RyR1 + IBMX**	**RyR1 + enprofylline**
State	primed	open	primed	open	primed	primed
PDB ID	9E18	9E19	9E1A	9E1B	9E1C	9E1D
EMDB ID	EMD-47385	EMD-47386	EMD-47387	EMD-47388	EMD-47389	EMD-47390
EMDB ID – focused map	EMD-47396	EMD-47397	EMD-47398	EMD-47399	EMD-47400	EMD-47401
**Data collection**						
Microscope	FEI Titan Krios		FEI Titan Krios		FEI Titan Krios	FEI Titan Krios
Detector	Gatan K3		Gatan K3		Gatan K3	Gatan K3
Voltage (kV)	300		300		300	300
Magnification	105,000		105,000		105,000	105,000
Exposure ($e^-/Å^2$)	58		58		58	58
Defocus range (μm)	-0.5 to -1.5		-0.5 to -1.5		-0.5 to -1.5	-0.5 to -1.5
Pixel size (Å)	0.83		0.83		0.83	0.83
**Processing**						
Software	cryoSPARC		cryoSPARC		cryoSPARC	cryoSPARC
Symmetry	C4		C4		C4	C4
Total particles (N)	67,689		130,467		128,983	94,090
Final particles (N)	64,353	3,336	113,183	17,284	128,983	94,090
Global map resolution (Å)*	2.68	4.04	3.35	4.49	2.63	2.76
Local map resolution (Å)[‡]	2.35	3.49	3.14	4.16	2.34	2.42
**Model Composition**						
Peptide chains	8	8	8	8	8	8
Nonhydrogen	144,144	144,236	144,128	144,128	144,120	144,112
Protein residues	18,044	18,044	18,044	18,044	18,044	18,044
Ligands	16	16	16	16	16	16
**Mean *B* factors ($Å^2$)**						
Protein	91.28	182.04	150.50	239.31	79.30	133.06
Ligands	40.65	67.56	85.13	114.25	64.50	69.85
**R.m.s. deviations**						
Bond length (Å)	0.013	0.004	0.005	0.004	0.005	0.004
Bond angles (°)	1.281	0.845	0.920	0.942	0.871	0.790
**Ramachandran**						
Favored (%)	97.32	97.15	97.02	96.90	96.74	98.19
Allowed (%)	2.50	2.76	2.89	2.97	3.17	1.81
Disallowed (%)	0.18	0.09	0.09	0.13	0.09	0.00
**Validation**						
MolProbity score	1.79	1.99	2.01	2.46	1.80	1.94
Clashscore	14.39	21.24	22.36	31.77	12.21	15.77
Rotamer outliers (%)	0.37	1.04	0.54	2.38	0.38	2.08
FSC model 0.5 (Å)[#]	2.84	6.29	3.85	6.06	2.78	2.98

For 7UA4 and 9E17, the only changes introduced were the orientation and position of the xanthine and caffeine molecules, respectively. The cryo-EM parameters can be found on the original publications of PDB 7TZC[19] (for PDB 9E17) and PDB 7UA4[24] (for its updated version).

Table S1 continuation.

Sample	RyR1 + uracil	RyR1 + allopurinol	RyR1 + oxypurinol	RyR1 + oxypyricid	
State	primed	primed	primed	primed	open
PDB ID	9E1E	9E1F	9E1G	9E1H	9E1I
EMDB ID	EMD-47391	EMD-47392	EMD-47393	EMD-47394	EMD-47395
EMDB ID – focused map	EMD-47402	EMD-47403	EMD-47404	EMD-47405	EMD-47406
**Data collection**					
Microscope	FEI Titan Krios	FEI Titan Krios	FEI Titan Krios	FEI Titan Krios	
Detector	Gatan K3	Gatan K3	Gatan K3	Gatan K3	
Voltage (kV)	300	300	300	300	
Magnification	105,000	105,000	105,000	105,000	
Exposure ($e^-/Å^2$)	58	58	58	58	
Defocus range (μm)	-0.5 to -1.5	-0.5 to -1.5	-0.5 to -1.5	-0.5 to -1.5	
Pixel size (Å)	0.83	0.83	0.83	0.83	
**Processing**					
Software	cryoSPARC	cryoSPARC	cryoSPARC	cryoSPARC	
Symmetry	C4	C4	C4	C4	
Total particles (N)	33,584	41,370	17,393	47,775	
Final particles (N)	33,584	41,370	17,393	14,467	33,308
Global map resolution (Å)*	2.92	3.03	3.17	3.26	3.20
Local maps resolution range (Å)‡	2.47	2.56	2.69	2.64	2.65
**Model Composition**					
Peptide chains	8	8	8	8	8
Nonhydrogen	144,088	144,104	144,104	144,116	144,120
Protein residues	18,044	18,044	18,044	18,044	18,044
Ligands	16	16	16	16	16
**Mean *B* factors ($Å^2$)**					
Protein	100.45	122.14	110.90	135.29	115.91
Ligands	98.17	80.35	69.09	73.11	73.58
**RMSD**					
Bond length (Å)	0.004	0.005	0.007	0.006	0.003
Bond angles (°)	0.622	0.676	0.695	0.658	0.670
**Ramachandran**					
Favored (%)	97.12	96.65	96.85	96.89	96.74
Allowed (%)	2.88	3.35	3.15	3.11	3.17
Disallowed (%)	0.00	0.00	0.00	0.00	0.09
**Validation**					
MolProbity score	1.61	1.72	1.66	1.67	1.80
Clashscore	8.35	9.70	8.75	9.16	10.11
Rotamer outliers (%)	0.68	0.71	0.83	0.64	1.23
FSC model 0.5 (Å)#	2.87	2.95	3.05	3.09	3.06

* Map resolution of the non-uniform refinement determined by CryoSPARC before local masks.

‡ Map resolution range represents the range determined by local refinements in cryoSPARC using the masks described in Methods.

\# Value obtained from Phenix validation tool.
